# Fitting-Free Diagnosis of Conduction-Model Breakdown in Laser Powder Bed Fusion

**DOI:** 10.3390/ma19153290

**Published:** 2026-08-03

**Authors:** Gisuk Hong, Jaebong Cho, Hyunbo Cho

**Affiliations:** Department of Industrial and Management Engineering, Pohang University of Science and Technology, 77 Cheongam-ro, Nam-gu, Pohang 37673, Republic of Korea; kisuk13577@postech.ac.kr (G.H.); jaycho00@postech.ac.kr (J.C.)

**Keywords:** laser powder bed fusion, keyhole transition, conduction model, additive manufacturing, process qualification

## Abstract

Melt-pool depth governs interlayer bonding and porosity in laser powder bed fusion and underpins part qualification, yet predicting it reliably remains difficult. Fast conduction models reach useful accuracy only after the absorptivity is fitted to the depths they are meant to predict, and inverse analyses have been used the same way, to recover a calibrated parameter rather than to test the model. Here, the absorptivity is fixed independently instead, a measured coupling for IN718 and, for IN625 and 316L, a published closed-form relation never fitted to the present depths. This converts a moving-source conduction model from an object of calibration into one of validation. The melt boundary is located by root-finding rather than on a grid, so no discretization error enters the diagnosis. Across 231 single tracks, the model reproduces conduction-regime depth and half-width to within a few percent and underpredicts increasingly once keyholing begins. Inverting each measured depth for the absorptivity conduction would require yielding a fitting-free diagnosis: no conduction-regime track demands a non-physical value, and the inferred value converges near 0.38 against inputs of 0.27 to 0.34, whereas every keyhole-classified track demands a value above unity. Because an inferred absorptivity also absorbs unmodeled transport, downward convection was emulated as an anisotropic effective diffusivity; at the enhancement reported for Marangoni flow, no keyhole track becomes explicable. A measured Ti-6Al-4V absorptivity rise of a factor 1.9 supports the mechanism. An enthalpy-indexed correction and data-driven baselines remain alloy-specific, whereas the physics-based model retains its advantage under cross-alloy extrapolation. All findings are for single tracks on bare plates.

## 1. Introduction

Laser powder bed fusion (LPBF) builds metal parts by scanning a focused laser over thin layers of powder, and it now serves the aerospace, energy, and biomedical sectors, where it enables part consolidation and design freedom beyond the reach of conventional manufacturing [1]. The laser melts a small pool that solidifies as the beam advances, and the depth of this pool determines whether successive layers fuse fully and whether the melt collapses into a keyhole, a deep vapor cavity that traps gas porosity and shortens fatigue life [2,3]. Reliable prediction of melt-pool depth is therefore a prerequisite for selecting process parameters and qualifying parts, and it remains a central modeling problem in metal additive manufacturing [4].

Two regimes govern the geometry of the pool. Below a threshold energy density, heat conduction sets the pool shape, and the depth and width grow together; above the threshold, the beam opens a vapor depression whose walls trap the beam through multiple reflections and drive the pool far deeper than conduction alone can account for. This is the keyhole regime [5], and the variable that distinguishes it from the conduction regime is the absorbed energy density, expressed through the normalized enthalpy of King et al. [5] and through closely related scaling parameters introduced by Fabbro [6] and by Rubenchik et al. [7].

Analytical descriptions of the pool itself rest on a small set of closed-form solutions. The Rosenthal point source [8] and its Gaussian-distributed extension by Eagar and Tsai [9] remain the basis of most fast models, while finite-element practice more often uses a volumetric Gauss–Goldak source that deposits energy over a prescribed depth [10]. These solutions reproduce the lateral extent of the pool well and its penetration poorly, and the reason is structural: a surface source produces a near-semicircular conduction pool, so the depth-to-half-width ratio it can attain is bounded close to unity. That bound is itself the basis of the conventional geometric criterion, under which a cross-section is taken to be conduction-dominated below an aspect ratio of one and keyholing above it [10]. Identification of the transition has otherwise proceeded along two lines. Energy criteria place a threshold on the normalized enthalpy [5] or on the related scaling groups [6,7], but the threshold is calibrated on measurements and shifts between datasets. Direct observation, by in situ X-ray imaging of the vapor depression or by operando absorptivity measurement [11,12], settles individual conditions but requires synchrotron or calorimetric access and therefore covers few parameter sets. Neither line answers the question a modeler actually faces, which is whether a given conduction model can reach a measured depth at all.

Two obstacles separate these scaling arguments from a quantitative prediction. First, the absorptivity that sets the energy input is itself regime-dependent and is rarely known in advance, so an analytical model must either assume a value or fit one to the same measurements it is intended to predict. Second, a surface conduction model contains none of the cavity physics that deepens the keyhole, so any depth it underpredicts could reflect either an underestimated absorptivity or a structural inadequacy of the model, and these two explanations are ordinarily indistinguishable. How accurately a physics-based model reproduces depth once its absorptivity is fixed to a correct value, and whether a residual error reflects the input or the model, is therefore the question that determines how far such a model can be trusted.

Most quantitative approaches to depth prediction adjust the model until it matches the measurements in one of two ways. The first is inverse analysis, in which the absorptivity is treated as a free parameter inferred from the measured depths. Whalen et al. [13], for example, calibrated an Eagar–Tsai model by Bayesian inference and reproduced the keyhole regime, but their inferred absorptivity rose toward unity at high power while a fitted porosity term absorbed the unmodeled vapor depression, so the recovered accuracy reflects the calibration rather than the underlying physics. The inversion performed here runs in the opposite direction: the absorptivity is never inferred as a model input, and the inferred value is read as a diagnostic of the model rather than used to repair it. The second approach learns depth directly from data, using Gaussian-process surrogates [14] or neural networks trained on high-fidelity temperature fields [15,16]. Such models can be accurate within the range on which they are trained, yet they inherit the cost or bias of their reference data and leave unanswered how a physics-based model behaves when its input is fixed to a correct value. The account that generalizes across the most alloys to date is that of Ye et al. [17], who collapsed the normalized depths of several alloys onto a single curve once the measured absorptivity was supplied. That result characterizes the measured data rather than testing a predictive model across the transition, yet it motivates the present approach by demonstrating that a correctly chosen absorptivity can unify chemically distinct materials.

A complementary line of work, to which the present study belongs, benchmarks closed-form scaling laws against standardized measurements. Naderi et al. [18] tested three established scaling laws against cross-sectional melt-pool measurements for nickel superalloys and found strongly regime-dependent errors; one widely used correlation underpredicted keyhole depth by more than 90%, an error that fell to about 35% only after a non-constant absorption term was introduced. Such benchmarking establishes where the breakdown occurs, but neither recovers the lost depth nor explains its cause. A parallel asymmetry appears in conduction models. An analytical heat-conduction model for the related process of wire-arc directed energy deposition [19] and conduction models for laser powder bed fusion [20] reproduce the lateral extent of the pool but mispredict its penetration once keyholing begins. High-fidelity thermo-fluid simulation captures the recoil and Marangoni dynamics responsible for this asymmetry, but at a cost of roughly 20 h on 64 cores per track [21], and this expense is precisely why fast analytical models remain in routine use. What the field still lacks is an inexpensive, physics-based test that determines whether the depth error can be corrected, what physically causes it, and whether any such conclusion holds across chemically distinct alloys.

Here, we address these questions by fixing the absorptivity to an independently determined value and evaluating a moving-source conduction model without any further adjustment. The absorptivity is an in situ coupling measurement for IN718 and the closed-form value of Ye et al. [17] for IN625 and 316L. Fixing the input converts the model from an object of calibration into an object of validation, which is what makes the three questions answerable. We first establish the model’s range of validity across three alloys that span the full conduction-to-keyhole transition. We then diagnose the physical cause of its failure by inverting each measured depth without bounding the absorptivity, a test that takes no absorptivity as input and therefore cannot be biased by one. We finally ask whether the depth error can be removed by a parameterized correction, and how the physics-based model compares with data-driven surrogates under interpolation and extrapolation. The inversion shows that no physical absorptivity lets surface conduction reach the measured depth once keyholing begins in both alloys to which it is applied. Because an inferred absorptivity absorbs any unmodeled transport, as well as optical absorption, we test that reading explicitly against convective deepening rather than assuming it away. An enthalpy-indexed correction recovers depth only within a single alloy. The physics-based model retains its advantage where it matters most, namely extrapolation between alloys. The novelty of this work lies in three steps. The first is to fix the absorptivity from independent data so that the inversion tests the model instead of calibrating it. The second is to treat the non-existence of any physical solution as a signal that requires no absorptivity input and therefore cannot be biased by one. The third is that this energy-based signal, computed from the measured depth alone, lands on the same boundary as the geometric criterion, which is computed from depth and width; the two are consistent readings of one conduction limit rather than independent confirmations of it, and we say so. The scope is bounded accordingly. All conditions are single tracks on bare substrates, so powder-induced changes in absorptivity and layer-to-layer heat accumulation lie outside the tests reported here, and the nickel datasets comprise seven and fourteen conditions against two hundred and ten for 316L. The entire analysis is released as a single notebook that reproduces every result from public benchmark data, with no finite-element or computational-fluid-dynamics solver.

## 2. Materials and Methods

### 2.1. Analytical Conduction Model

The melt pool is modeled as a Gaussian laser source that translates at constant speed over a semi-infinite solid. The quasi-steady temperature field follows the Eagar–Tsai extension [9] of the Rosenthal solution [8]. The temperature rise at a point (x, y, z) follows from integrating the moving point-source response over the dwell history.

(1) $$T(x,\;y,\;z)=T_{0}+\frac{A\;P}{\rho \;c_{p}\;{\left(2\pi \right)}^{3/2}}\int_{0}^{\infty }g(\tau^{\prime })\;d\tau^{\prime }$$

The kernel, *g*, carries the Gaussian spreading and the depth decay of the moving source.

(2) $$g(\tau^{\prime })=\frac{exp\left[-\frac{{\left(x+v\tau^{\prime }\right)}^{2}+y^{2}}{2\left(\sigma^{2}+2a\tau^{\prime }\right)}-\frac{z^{2}}{4a\tau^{\prime }}\right]}{\left(\sigma^{2}+2a\tau^{\prime }\right){\left(2a\tau^{\prime }\right)}^{1/2}}$$

Here, *A* is the absorptivity, *P* the laser power, and v the scan speed. The thermal diffusivity, *a*, is the ratio of the effective conductivity to the volumetric heat capacity. The Gaussian source parameter, σ, is one quarter of the measured D4σ beam diameter. The integral is evaluated numerically. The melt-pool boundary is the liquidus isotherm, and its depth and half-width are located by root-finding on the isotherm itself rather than by scanning a spatial grid, so the boundary carries no discretization error in either direction; predicted and measured widths are compared as half-widths, consistent with the aspect-ratio definition. The predicted half-width is the maximum over the trailing length of the pool, which occurs at the surface; evaluating it on the beam centerline instead understates it by a margin that grows with pool depth. As σ approaches zero, the surface solution reduces to the Rosenthal point source, and we use this limit to verify the implementation. The thermophysical inputs are listed in Table 1. Conductivity and specific heat are averaged over the solidus-to-liquidus interval, and the latent heat of fusion is folded into the heat content that defines the normalized enthalpy below. Because the moving-source field carries sensible heat only, neglecting the latent-heat sink at the solid–liquid front can only overpredict the pool. It therefore cannot produce the observed depth deficit or the above-unity inversion. The conductivity and specific heat are temperature dependent, and values averaged over the melting range are an approximation. To bound the error this introduces, the IN718 prediction was repeated with property pairs taken at 500, 700, and 1350 C from tabulated data, a range that spans a factor of two in conductivity; the predicted depth changes by between −3.1 and +6.8 percent, roughly one seventh of the keyhole deficit it would need to explain. The melting-range averages implied by the same data, 28.6 W m^−1^ K^−1^ and 666 J kg^−1^ K^−1^, agree with the values listed in Table 1. The robustness analysis in Section 3.4 shows, in addition, that a 10% change in these properties leaves the keyhole-necessary fraction unchanged.

### 2.2. Effective Transport, Absorptivity Input, and Keyhole Indicator

Throughout, fitting-free means that no quantity is adjusted against the depth measurements analyzed here; the closed-form absorptivity relation used for IN625 and 316L is an empirical correlation established on independent data. A surface conduction model omits convection inside the molten pool, so the effective conductivity that reproduces a real pool can exceed the quiescent value. We do not calibrate this quantity for any alloy. For all three alloys, we fix the conductivity at the physical reference value listed in Table 1, and melt convection is invoked only as the reason an effective conductivity replaces the quiescent one [22]. The predicted width constrains the conductivity only weakly, changing by about ten micrometers across the plausible conductivity range, so a width-based calibration cannot determine it. We therefore use the width as a loose consistency check and retain the depth, which is never adjusted, as the quantity that tests the model. This choice also prevents the conductivity from being confounded with the closed-form absorptivity used for IN625 and 316L. A robustness analysis confirms that the central diagnosis does not depend on the conductivity value.

Where no in situ coupling measurement is public, the absorptivity is taken from the closed-form scaling of Ye et al. [17], which describes the effective absorptivity as an asymptotic exponential in the normalized enthalpy that saturates at 0.70 with a decay constant of 0.66. Below the transition, the absorptivity is held at the flat-surface value tabulated by the same authors for each alloy, 0.27 for IN625 and 0.34 for 316L; these are independent calorimetric values and are not adjusted here. The transition itself is indexed by the normalized enthalpy of King et al. [5].

(3) $$\beta =\frac{A\;P}{\rho \;h_{s}\;\sqrt{\pi \;a\;v\;\sigma^{3}}},\;\;\;\;\;\;h_{s}=\rho \;c_{p}\;\left(T_{l}-T_{0}\right)$$

Here, *h_s_* is the heat content required to reach melting, including the latent heat, and *T_l_* is the liquidus temperature. We also report the aspect ratio, *d/(w/2)*. The conduction limit of one in this convention is not an arbitrary choice: a Gaussian surface source produces a near-semicircular conduction pool, and the source of the 316L data adopts the same limit on that basis [10]. Because the source dataset states its criterion as depth over full width, we report both conventions, in which conduction, transition, and keyholing correspond to *d/(w/2)* below one, between one and two, and above two. Regime statistics follow this three-way classification rather than a single threshold, so that no condition is discarded as ambiguous.

### 2.3. Benchmark Data

The model is applied to single-track measurements for three alloys, summarized in Table 2. For IN718, the NIST AM-Bench AMB2022-03 dataset [23] gives depth and width with an in situ measurement of dynamic laser coupling for 7 conditions. The laser power ranges from 245 to 325 W, the scan speed from 800 to 1200 mm s^−1^, and the D4σ spot from 49 to 82 µm, and the measured coupling ranges from 0.55 to 0.73. This coupling supplies the independent absorptivity that enables fitting-free validation and inversion, and the dataset also reports the standard deviation of six repeat cross-sections per condition, which is used as the measurement uncertainty in Section 3.1. For IN625, 14 single-track conditions on the same testbed [24] sweep the spot diameter from 50 to 256 micrometers at two power levels, 180 and 195 W, thus moving the pool from keyhole melting at the smallest spot to conduction melting at the largest. For 316L stainless steel, a single-source dataset of bare-plate tracks [10] gives 210 conditions in which power, scan speed, and spot size vary together. No in situ coupling measurement is public for IN625 or 316L, so their absorptivity is the closed-form value described above. The source of the 316L data does not state where in the cross-section the width is measured, nor does it report repeat measurements or a measurement uncertainty, so 316L widths are reported for consistency only, and the depth carries the validation for that alloy. The nominal spot diameters of that dataset are set by defocusing, and their beam convention is likewise unstated; we assume they are D4-sigma values and report the sensitivity of the diagnosis to that assumption in Section 3.4.

A fourth alloy supports the absorptivity input rather than the depth analysis. The NIST A-AMB2022-01 dataset [11] reports time-resolved absorptance for Ti-6Al-4V bare plates under a stationary laser pulse and a scanned track, measured by integrating-sphere radiometry. The depth in this dataset is recorded only in high-speed X-ray images and is measured in situ rather than by post-solidification metallography. These two facts make the Ti-6Al-4V depth incompatible with the cross-sectional liquidus depth of the other three alloys, so we do not place it on the same axis. We use Ti-6Al-4V only for its measured absorptivity, which gives a second alloy with a directly measured input.

### 2.4. Validation, Depth Recovery, and Inversion

The measured or closed-form absorptivity is supplied directly to Equation (1), and depth and width are predicted with no adjustment of the input. We define the depth deficit as the measured depth minus the predicted depth, and we index it on the normalized enthalpy. A recovered depth adds one correction term to the conduction baseline.

(4) $$d_{hybrid}=d_{cond}+\delta (\beta ),\;\;\;\;\;\;\delta (\beta )=c_{1}\beta +c_{0}$$

The two coefficients are fitted by least squares, either within one alloy or on the pooled data of all three. Generalization is assessed by leave-one-out cross-validation within each alloy and by five-fold cross-validation on the pooled set. The correction is an empirical recovery, not a first-principles keyhole model.

**Figure 1:**
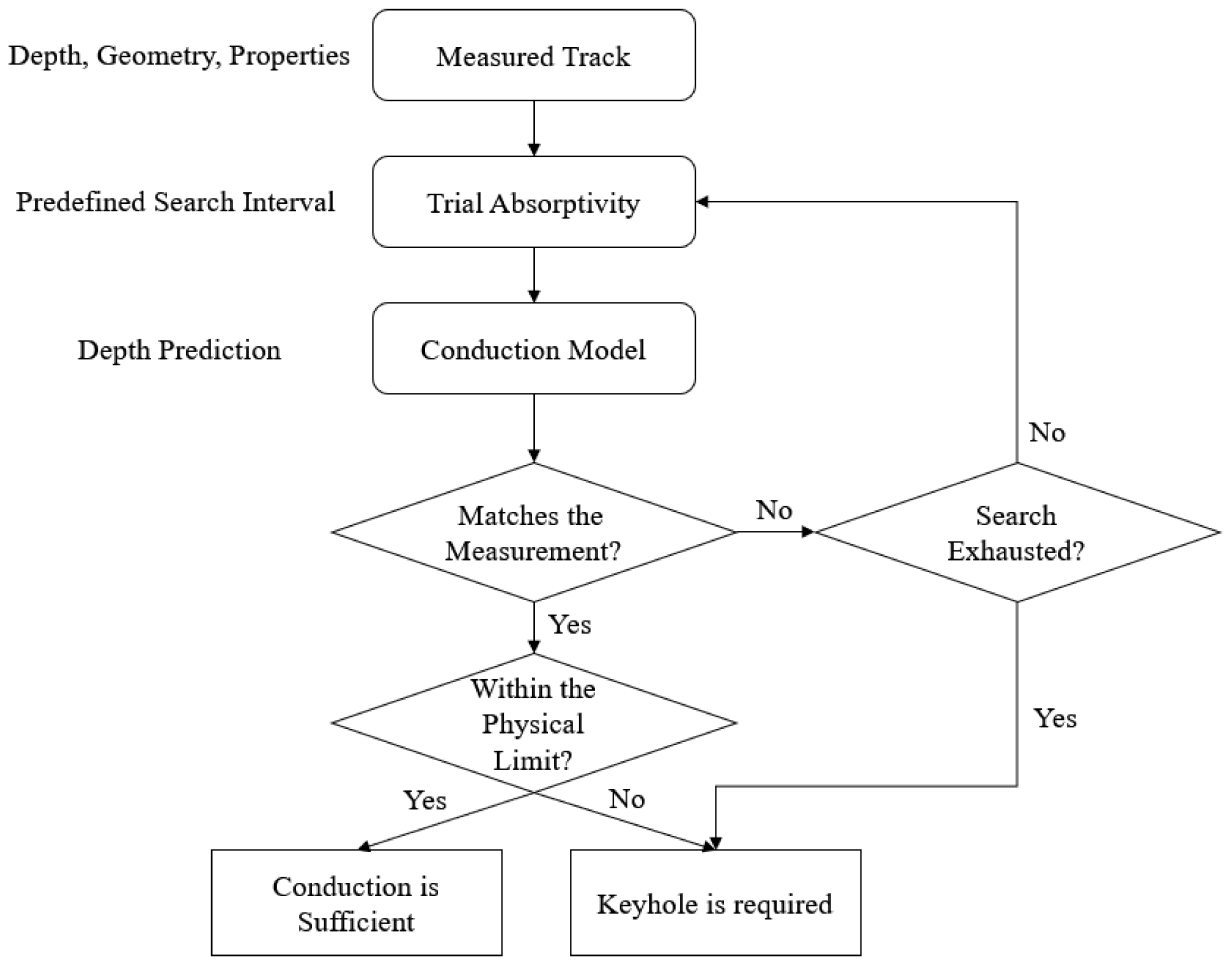
The fitting-free inverse analysis. A trial absorptivity is adjusted until the conduction model reproduces the measured depth; the converged value is then compared with the physical limit. Conditions for which no value in the interval reproduces the measured depth reach the same verdict as those exceeding the limit.

The central method is an unconstrained inversion of the forward map. Figure 1 summarizes the procedure and its three termination paths. For each condition, we find the absorptivity that reproduces the measured depth under pure conduction.

(5) $$A_{cond}=\arg\underset{A}{\min}\left|\;d_{model}(A)-d_{meas}\;\right|$$

The problem is solved by Brent’s method on the interval from 0.02 to 8, with no upper physical bound imposed on the solution and an absorptivity tolerance of 2 × 10^−3^, and the iteration terminating when the bracketing interval falls below that tolerance. The upper limit of 8 lies far above any physical absorptivity, which cannot exceed unity, so a condition that admits no solution below 8 cannot be reproduced by any physically attainable absorptivity. Only the measured depth enters, so the result is independent of the absorptivity assumed in the forward model. A value below unity means that conduction with a physical absorptivity can account for the depth. A value above unity, or one unreachable anywhere on the search interval, means that it cannot, so keyhole penetration is required.

**Figure 2:**
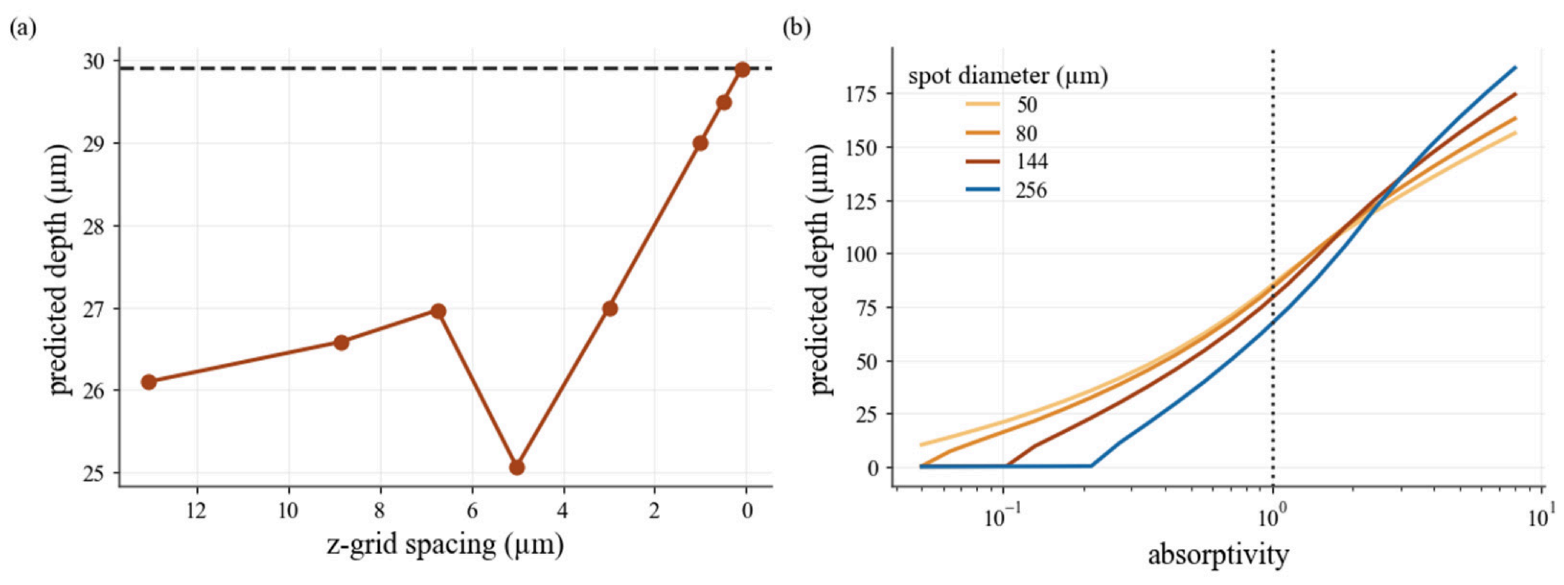
Numerical verification. (**a**) Predicted depth when the isotherm is snapped to a z-grid; the dashed line is the grid-free solution. (**b**) Depth against absorptivity at four spot diameters, showing that the inverse solution is unique.

Two properties make the inversion well posed. First, for a fixed geometry, the predicted depth increases strictly with the absorptivity, because more absorbed power can only deepen the pool. The map from absorptivity to depth is therefore monotone, and its inverse is unique wherever it exists below the physical ceiling. We confirm this numerically at four spot diameters spanning the datasets, finding that the predicted depth rises without exception as the absorptivity increases across the search interval (Figure 2b). Second, when the measured depth exceeds the deepest pool the model can produce at the ceiling, no solution exists. This non-existence is itself the signal of keyhole necessity, not a numerical failure.

### 2.5. Computational Setup

Because the melt boundary is located by root-finding rather than snapped to a grid, no discretization error enters the depth or the half-width. The remaining discretization is the sampling of the trailing length and of the dwell integral in Equation (1); both are converged to five significant figures at 41 and 1000 points, respectively, and the vanishing-spot limit reproduces the Rosenthal solution to within 0.1 percent on the surface centerline. For comparison, locating the boundary on a z-grid of the spacing used in common practice biases the predicted depth by 10 to 16 percent, and the bias is not monotone in the grid spacing (Figure 2a). The keyhole diagnosis is repeated at three values of the effective conductivity for each closed-form alloy, namely 20, 36, and 50 W m^−1^ K^−1^ for 316L, and 20, 25, and 30 W m^−1^ K^−1^ for IN625. The robustness tests perturb the conductivity by 10% in either direction, the specific heat upward by 10%, and the liquidus temperature upward by 5%.

Three data-driven baselines provide a comparison for the recovery. They are support-vector regression with a radial-basis kernel, a feedforward network with two hidden layers of 32 units, and Gaussian-process regression with a radial-basis-plus-noise kernel. Each receives the normalized enthalpy and the conduction-model depth as inputs and predicts the measured depth; inputs and targets are standardized before training. Hyperparameters are selected inside the cross-validation loop by grid search on a four-fold inner split, so no test condition informs model selection. The grids cover the penalty, kernel width and tolerance of the support-vector model, the hidden-layer size and weight decay of the network, and the kernel length scale and noise level of the Gaussian process; the modal selections are reported with the results. The baselines are evaluated under interpolation by leave-one-out cross-validation across the pooled nickel-alloy conditions and under extrapolation by training on IN625 and predicting IN718. The multivariable correction uses a random-forest regressor of up to 300 trees with depth capped at 6, with four process descriptors as inputs. These are the normalized enthalpy of Equation (3): a volumetric energy density, defined as the power divided by the product of scan speed and squared beam diameter; a Péclet number, defined as the scan speed times the beam diameter divided by the thermal diffusivity; and the beam dwell time, defined as the beam diameter divided by the scan speed. The correction is evaluated by five-fold cross-validation within the pooled data and by leave-one-material-out cross-validation across alloys.

## 3. Results

### 3.1. Range of Validity Under a Fixed Input

**Figure 3:**
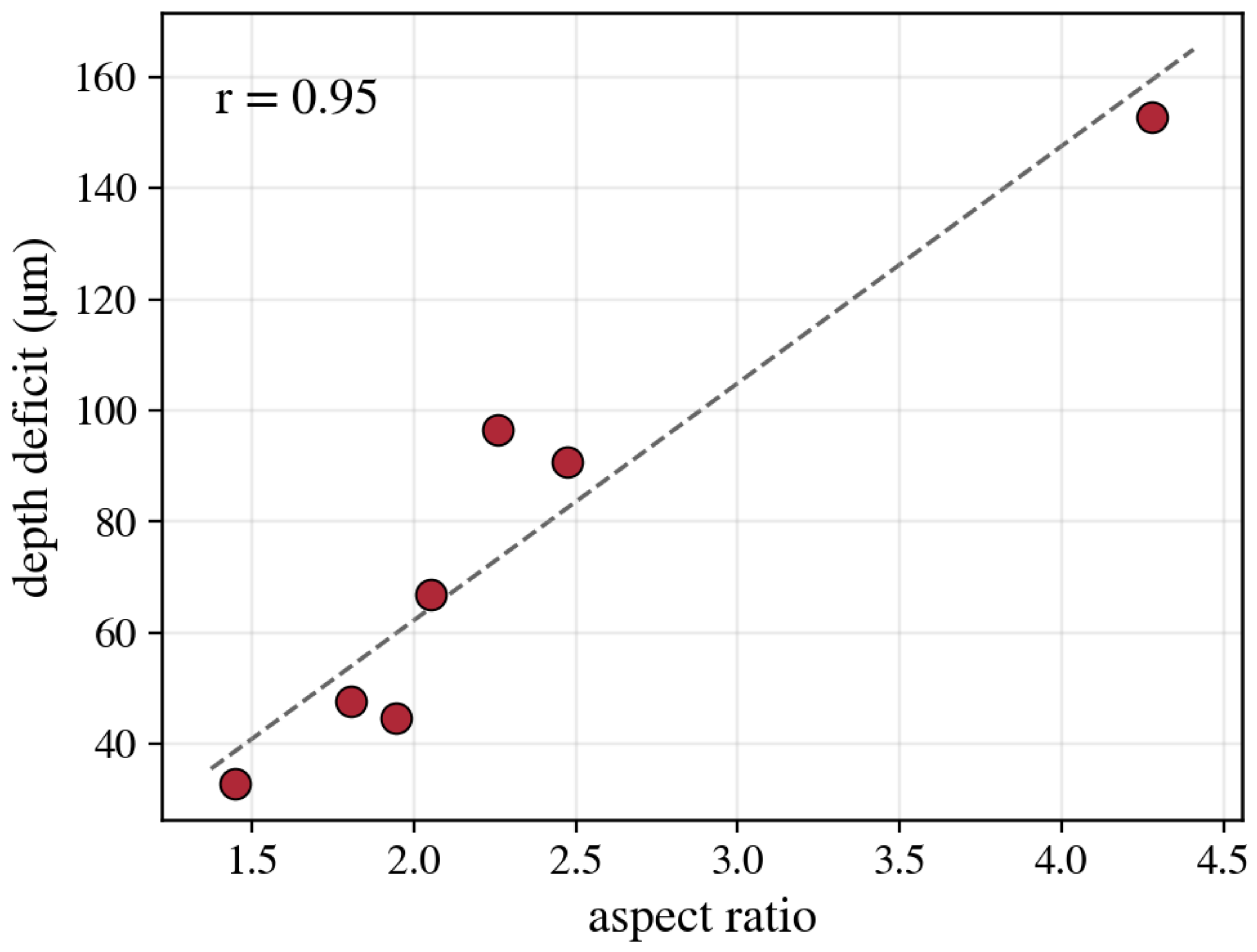
IN718 depth deficit versus aspect ratio (*r* = 0.95); dashed line is a linear fit.

With the absorptivity supplied directly, the conduction model distributes the absorbed energy laterally rather than downward. For IN718, the depth is underpredicted in all seven conditions, by 46 percent on average, while the predicted half-width exceeds the measured value by 24 percent. The two errors have opposite sign, which is the expected signature of a pool that has ceased to be a conduction cap. The deficit grows with the aspect ratio, and the sample correlation is 0.955 (Figure 3). Relative to the scatter of six repeat cross-sections per condition, the depth deviation is larger by a factor of about 35, and the half-width deviation by a factor of about 12, so neither can be attributed to measurement error. Because the IN718 set contains only seven conditions, this trend is indicative rather than definitive, and the larger IN625 and 316L datasets carry the statistical weight. For 316L, which supplies 210 of the 231 conditions, the conduction regime is reproduced closely: the mean depth error is +1.5 percent, and the mean half-width error is +1.1 percent, so depth and width are matched simultaneously without any adjustment. Across the keyhole conditions of the same alloy, the depth is underpredicted by 56 percent. The IN625 conduction conditions behave differently, with a mean depth error of −33 percent and a half-width overprediction of 40 percent. These ten conditions are the most strongly defocused in the study, with nominal spot diameters of 122 to 256 micrometers and depths of only 26 to 50 micrometers, so the ratio of depth to spot size falls to 0.15, and the melting is marginal. Section 3.4 shows that a beam 40 percent narrower than the nominal diameter reconciles depth and width simultaneously for these conditions, and that the keyhole diagnosis is unaffected either way; we therefore treat the large defocused spots as lying outside the range in which the Gaussian surface source is quantitatively reliable. Table 3 collects these figures. In all three alloys, the boundary between success and failure coincides with the keyhole transition, which indicates that the limitation is intrinsic to surface conduction rather than specific to one alloy.

**Figure 4:**
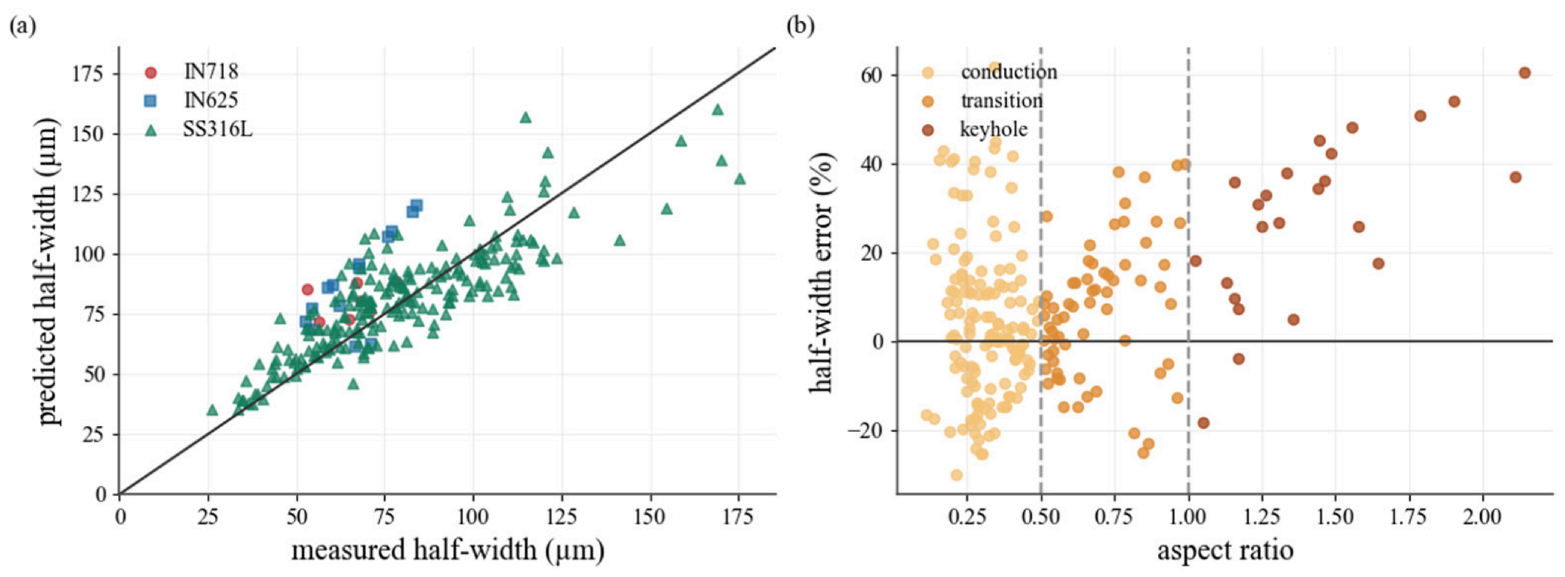
Half-width validation. (**a**) Predicted against measured half-width; bars are the IN718 measurement scatter. (**b**) Half-width error against aspect ratio; dashed verticals mark the conduction limit and the keyhole onset.

A consistency check supports the numerical implementation. As the beam size vanishes, the surface solution reproduces the Rosenthal point-source limit to within a fraction of a percent, which confirms the integration scheme. The three alloys all use the physical reference conductivity, with no per-alloy calibration. The half-width is matched in the conduction regime and increasingly overpredicted as the pool deepens, with the error rising from 1 to 27 percent between the two extremes (Figure 4), which is why it serves as a consistency check, while the depth, which is never adjusted, remains the quantity that tests the model.

### 3.2. Diagnosis of Keyhole Necessity

**Figure 5:**
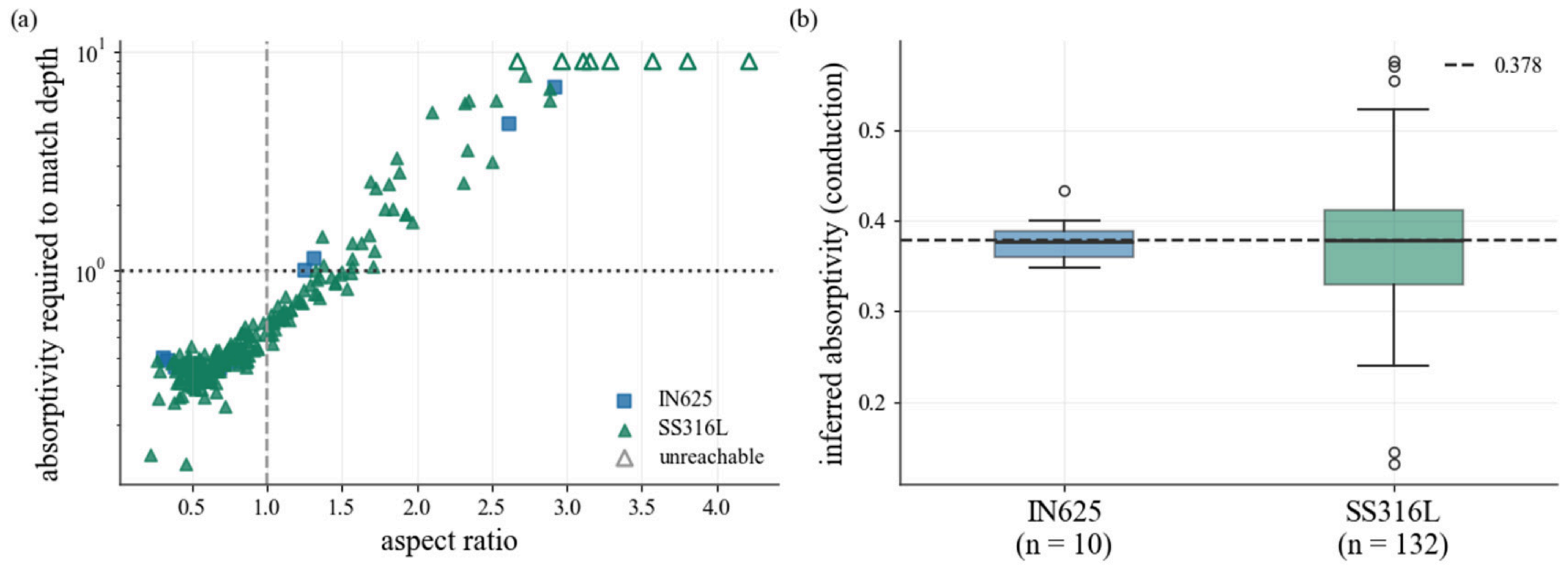
Inferred absorptivity for IN625 and 316L. (**a**) Value required to reproduce the measured depth; squares are IN625 and filled triangles 316L, open triangles mark conditions unreachable at 8, the dotted line marks the physical limit, and the vertical dashed line an aspect ratio of 1. (**b**) Conduction-regime values converge near 0.38 (dashed line), against closed-form inputs of 0.27 and 0.34; open circles are outliers.

The inversion of the measured depth identifies the physical cause of the depth shortfall, and it gives the same answer for chemically distinct alloys (Figure 2). The inversion is applied to IN625 and 316L, because IN718 already has a measured absorptivity and needs no inference. In the conduction regime, the inferred absorptivity is physically admissible. Its median is 0.377 for IN625 and 0.378 for 316L, against closed-form inputs of 0.27 and 0.34, respectively (Figure 5b). The two alloys agree with one another to three decimal places, although their inputs differ by a quarter, which is the more informative comparison, since the inversion is free to return any non-negative value. The residual gap to the input is not explained by a single effective conductivity. Solving for the conductivity that would reproduce each measured 316L conduction-regime depth at the closed-form absorptivity, 90 of the 132 conditions admit no solution anywhere between 3 and 400 W m^−1^ K^−1^, and the 42 that do span a factor of seven. An unmodeled transport enhancement therefore cannot be the whole account of the difference, and we do not claim it as one. The convergence is not imposed, since the inversion may return any non-negative value. The decisive point is that two chemically distinct alloys return the same physically reasonable absorptivity wherever conduction applies.

In the keyhole regime, the inferred effective absorptivity crosses the physical limit. Classified by the criterion of the source dataset, every one of the 24 keyhole conditions across the three alloys requires an absorptivity above unity, and 11 of them cannot be reproduced even at an absorptivity of 8. Under the more permissive threshold used in the submitted analysis, 37 of the 55 316L tracks above an aspect ratio of 1.2 require an above-unity value. For IN625, all four keyhole conditions require a value above unity, reaching 6.9 at the smallest spot, and for IN718, all seven conditions do, with three unreachable on the search interval (Figure 5a). Because a physical absorptivity cannot exceed unity, surface conduction cannot reach the measured depth, and a mechanism beyond surface conduction, consistent with keyholing, is required. Because the inversion uses only the measured depth and no absorptivity input, this statement is independent of the absorptivity assumed in the forward model, and it holds separately for each alloy to which the inversion is applied. It is a statement about what a surface-conduction description can reach, not yet a proof that keyholing is the only admissible mechanism; Section 3.4 tests the principal alternative. Whalen et al. [13] reproduced such depths by raising a fitted absorptivity toward unity. Once the absorptivity is left unconstrained, the same depths demand a value beyond unity, now in two alloys independently.

### 3.3. Coincidence with the Geometric Keyhole Threshold

**Figure 6:**
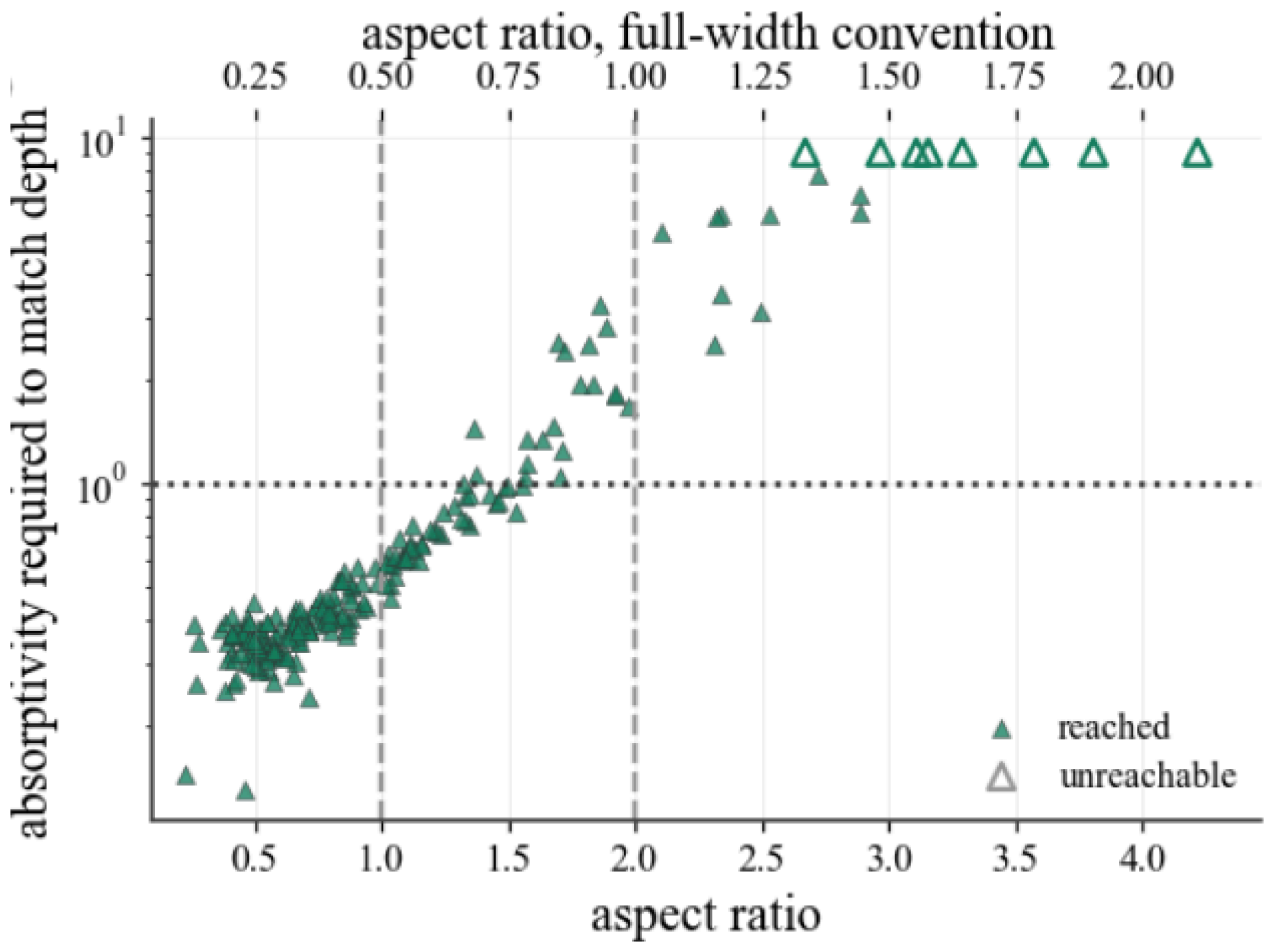
For 316L, the absorptivity required to reproduce depth (energy criterion, log scale) versus aspect ratio (geometric criterion). No track below 1.2 requires an above-unity value, and every track above 1.6 does. Filled triangles are reached tracks and open triangles conditions unreachable at 8; the dotted line marks the physical limit, the vertical dashed lines aspect ratios of 1 and 2, and the upper axis the full-width convention.

That the inferred absorptivity must cross the physical limit somewhere within the transition is unsurprising; the mechanistic test lies in where it crosses. The two criteria draw on different measured quantities: the geometric criterion uses depth and width together, whereas the inversion uses the measured depth alone. They are not, however, independent in origin. The geometric limit of one is itself derived from the shape a Gaussian surface source can produce, so the agreement below is a consistency check on that shared limit rather than a second and separate line of evidence, and we present it as such. We classify each 316L track by its measured aspect ratio, a purely geometric quantity, and separately by whether its inferred absorptivity exceeds unity, and the two classifications agree. The transition is sharp. Of the 155 tracks below an aspect ratio of 1.2, not one requires an above-unity absorptivity, and of the 32 tracks above 1.6, each one does; the change occurs within the narrow band between, while the inferred absorptivity climbs from a conduction-regime median of 0.378 to the ceiling of the search interval (Figure 6). The few keyhole-side tracks still reachable by conduction sit in the narrow band just above one, where geometry and energy are both marginal, which is the expected location of any disagreement.

This coincidence supplies a physical interpretation that a depth-error correction alone cannot: the conduction model fails abruptly at a threshold rather than degrading gradually, and that threshold marks the point at which the pool ceases to be a shallow conduction cap and becomes a deep cavity whose walls absorb the beam through multiple reflections. The inversion detects the same point as the place beyond which no attainable absorptivity can reach the measured depth, because that depth is no longer produced by surface conduction. The geometric criterion is an observation, namely that the pool is deeper than it is wide; the energy criterion is an inference, namely that surface conduction is insufficient. Their agreement across an alloy in which power, speed, and spot size vary independently shows that the inversion tracks the pool geometry rather than the parameterization, which is what a diagnostic built on a shape-limited forward model should do.

### 3.4. Robustness of the Diagnosis

Because the keyhole diagnosis is the central claim of this work, we examine its dependence on the model assumptions directly. Three classes of assumption could, in principle, manufacture the above-unity absorptivities: the thermal-property values, the effective conductivity that stands in for convection, and the closed-form absorptivity used where no measurement exists. None alters the conclusion, as Table 4 shows; the perturbations of 10% in conductivity and specific heat and 5% in liquidus temperature bracket the spread of reported values for these alloys. Resolved by regime, the diagnosis is unchanged at both ends: under every perturbation, no conduction-regime track requires an above-unity absorptivity, and every keyhole-classified track does, while the transition band moves between 32 and 42 percent. The tracks that admit no solution at all retain none under any perturbation. The above-unity absorptivities exceed unity by factors of up to 8, a gap that small property errors cannot close.

The effective conductivity is the most consequential assumption, because it sets the conduction depth directly. Varying it across the full physical range from 20 to 50 W m^−1^ K^−1^ leaves the keyhole-necessary fraction unchanged. The beam convention is a second assumption of the same kind, since the 316L and IN625 spot diameters are nominal. Halving the assumed Gaussian source parameter changes the conduction-regime inferred absorptivity substantially, from 0.378 to 0.548, but moves the keyhole-necessary fraction only from 67 to 71 percent; for IN625, the keyhole conditions require an above-unity value at every scaling between 0.5 and 1.0 of the nominal diameter, while a scaling of 0.6 brings the conduction-regime depth error to −0.1 percent and the half-width error to +7 percent simultaneously. The absolute value of the inferred absorptivity is therefore sensitive to the beam convention, and the diagnosis is not. The closed-form absorptivity does not enter the diagnosis at all, because the inversion solves for the absorptivity and cannot be biased by an assumed one. The conclusion rests on the non-existence of any solution, which is a qualitative fact rather than the precise value of any inferred quantity.

**Figure 7:**
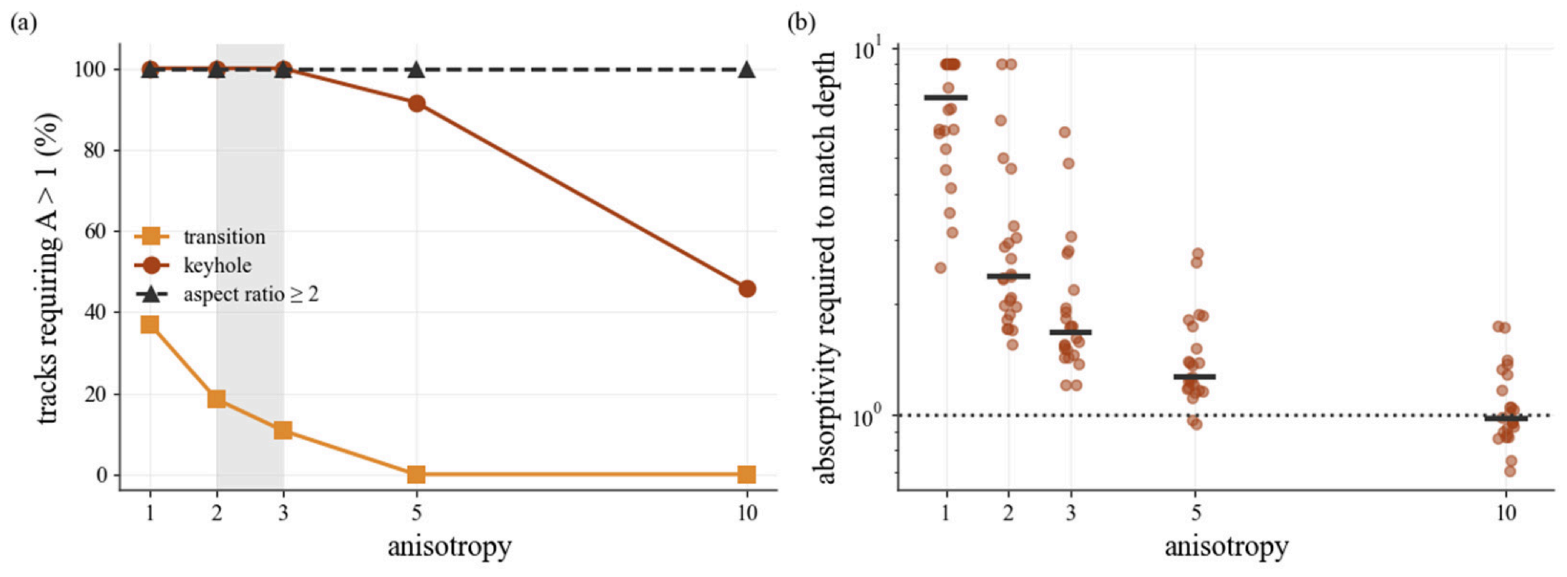
Effect of anisotropic effective transport, emulating downward advection. (**a**) Fraction of tracks requiring an above-unity absorptivity against the scaling factor; the shaded band is the range reported for Marangoni-driven flow, and the dashed line is the high-aspect-ratio subset. (**b**) Required absorptivity for the 24 keyhole-classified conditions, with medians as bars and the dotted line at unity.

The most substantive alternative is that convection, rather than a vapor cavity, delivers the missing depth. An isotropic effective conductivity cannot represent this, because downward advection is directional. We therefore repeated the inversion with an anisotropic effective diffusivity, scaling the through-thickness component by a factor while leaving the in-plane component fixed, which is the simplest continuum surrogate for a downward-biased flow. The attainable aspect ratio rises with this factor, from 0.96 in the isotropic case to 1.36 at a factor of two and 1.66 at a factor of three, so the geometric limit invoked above is a property of the isotropic assumption and not of conduction as such. The diagnosis nevertheless survives. Over the 89 transition and keyhole conditions, an enhancement of two to three, the range reported for Marangoni-driven transport, resolves part of the transition band but not one of the 24 keyhole-classified conditions. A factor of five still leaves 22 of the 24 requiring an above-unity absorptivity, a factor of ten leaves 11, and the seven conditions with the largest aspect ratios remain unreachable at a factor of ten and an absorptivity of eight. Convection of the strength reported in the literature therefore accounts for the transition band, where it is expected to matter, and not for the keyhole conditions (Figure 7).

**Figure 8:**
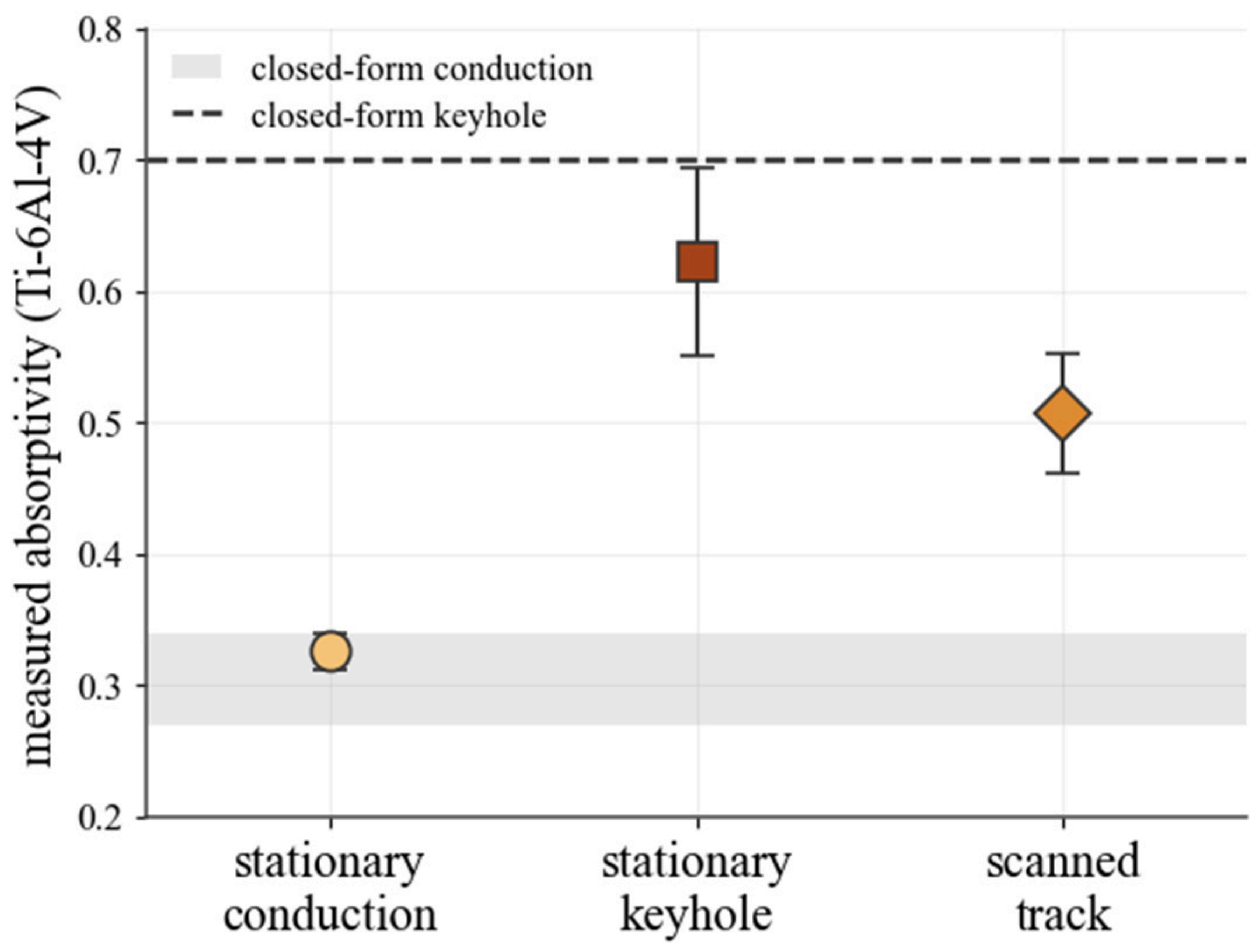
Measured Ti-6Al-4V absorptivity: Stationary-pulse conduction and keyhole windows (circle and square) and a scanned track (diamond) against the closed-form plateaus (dashed). Bars are the standard deviation of the instantaneous absorptivity within each window, a physical fluctuation rather than a measurement uncertainty. The rise across the transition is a factor of 1.9.

A second, independent check comes from a directly measured absorptivity. The diagnosis infers that the absorptivity must rise sharply at the keyhole transition. For IN625 and 316L, this rise is assumed through the closed-form input and is never measured. The Ti-6Al-4V data of Simonds et al. [11] provide the measurement. Under the stationary pulse, the measured absorptivity holds near 0.33 while the pool is shallow, and then it rises to about 0.62 once a keyhole forms, a factor of about 1.9. Because the conduction model used here is a moving-source model, we also report the absorptivity measured on a scanned track at 196 W and 700 mm/s; this single steady-state value of 0.51 lies between the two stationary plateaus, consistent with a track that straddles the transition (Figure 8). The conduction-regime value of 0.33 lies between the flat-surface and minimum absorptivities tabulated for this alloy by the same authors, 0.39 and 0.26, and is of the same magnitude as the plateaus used for the other two alloys, 0.27 and 0.34; the keyhole-regime value of 0.62 lies below the saturation value of 0.70. What transfers cleanly is therefore the size of the rise across the transition rather than agreement with any single plateau. The assumed conduction-to-keyhole rise is therefore observed directly in a fourth alloy. Two of the four alloys now carry a measured absorptivity, and these two anchor the closed-form input used for the other two (Table 2). The inference behind the diagnosis is consistent with direct measurement, not only with a closed-form assumption.

### 3.5. Material Specificity of the Depth Correction

**Figure 9:**
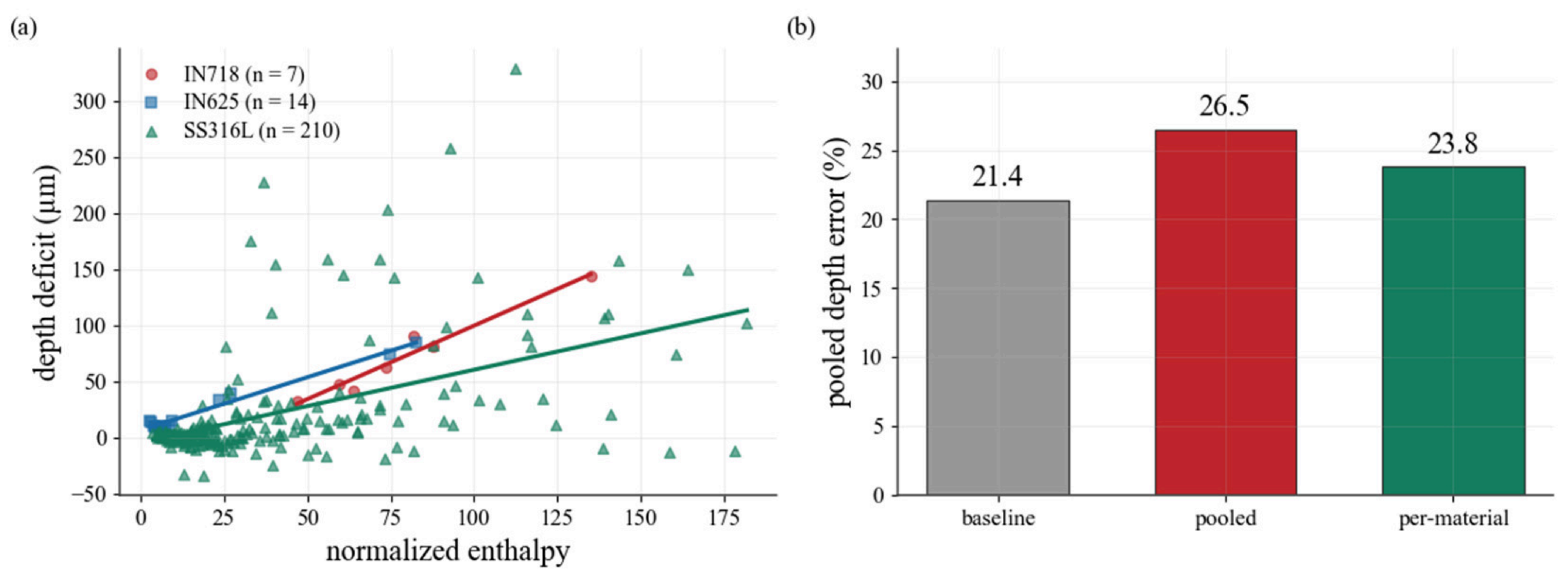
Depth deficit versus normalized enthalpy. (**a**) Per-alloy fits with diverging slopes; each solid line is the least-squares fit for the alloy of the same color. (**b**) Pooled depth error without correction, with a pooled fit, and with a per-alloy fit.

A correction indexed on the normalized enthalpy reduces the depth deficit, but only within one alloy. Fitted per alloy, it lowers the leave-one-out depth error, as shown in Table 5: the improvement is large for IN718 and moderate for IN625, whereas for 316L, the correction does not help, and the error stays at the uncorrected baseline. The reason is visible in the fitted slopes, which differ markedly between alloys, so that the deficits do not collapse onto a single line. Within each nickel alloy the deficit tracks the enthalpy tightly, with correlations of 0.979 and 0.987, whereas the 316L correlation is 0.486, and pooling the three alloys lowers the deficit-enthalpy correlation to 0.513 (Figure 9a). A single pooled correction consequently raises the pooled depth error from 21.4 to 26.5 percent rather than reducing it, and fitting per alloy leaves it at 23.8 percent (Figure 9b). The pooled figure is dominated by 316L, which supplies 210 of the 231 conditions and is the alloy the correction fails; taken alone, the correction lowers the IN718 error from 45.9 to 4.9 percent and the IN625 error from 37.3 to 8.0 percent, while raising the 316L error from 19.5 to 25.5 percent.

**Figure 10:**
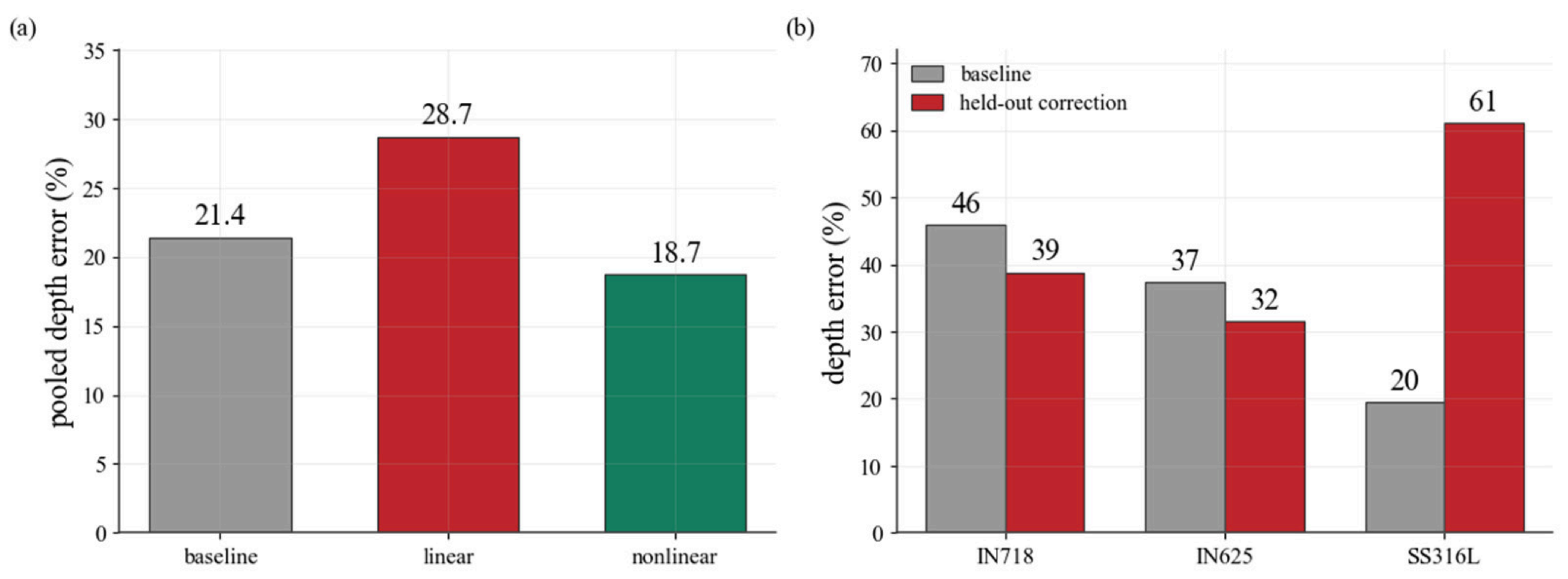
Multivariable correction. (**a**) Pooled five-fold cross-validation. (**b**) Leave-one-material-out cross-validation, where the correction fails to transfer.

A natural objection is that the normalized enthalpy is simply the wrong single variable, and that a richer correction would transfer across alloys. We tested this directly. Beyond the normalized enthalpy, we added the volumetric energy density [25], a Péclet number, the beam dwell time, and the conduction-model depth as candidate inputs, and we fitted both linear and nonlinear corrections. The volumetric energy density correlates with the deficit more strongly than the normalized enthalpy does, with a coefficient of 0.708 against 0.513, so the normalized enthalpy is not the best available single predictor. No linear combination of these variables beats the uncorrected baseline under cross-validation, which shows that the deficit depends on them nonlinearly. A nonlinear correction does reduce the pooled cross-validated error to 17.2 ± 0.7 percent, whereas a linear one raises it to 28.6 ± 0.2 percent (Figure 10a). The improvement does not transfer between alloys. When one alloy is held out of training entirely and then predicted, the nonlinear correction fails, and for 316L, it reaches 62 percent against that alloy’s own baseline of 19.5 percent (Figure 10b). Transferability follows regime overlap rather than chemical similarity, and it is asymmetric. A correction fitted on IN625 lowers the IN718 error from 45.9 to 11.0 percent, whereas the reverse direction raises the IN625 error to 78.3 percent, worse than applying no correction at all; every nickel-fitted correction degrades 316L, two thirds of whose conditions lie in the conduction regime where the deficit is near zero. The correction is an interpolator within a sampled regime and not a transferable law, whether it uses one variable or several, and whether it is linear or nonlinear. This reinforces the central contrast of the study: the inversion needs no fitted correction and transfers across alloys by construction, so the robust cross-material result is the diagnosis, not the correction.

### 3.6. Benchmark Against Data-Driven Baselines

The value of the physics-based backbone depends on whether the model interpolates within dense data or extrapolates beyond it. The pooled comparison in Table 6 uses the two nickel alloys, where the conditions are sampled one variable at a time and the enthalpy axis is well behaved. We exclude 316L from this pooled comparison for the same reason it breaks the pooled correction, namely its joint variation in power, speed, and spot size. Under nested leave-one-out cross-validation across the pooled nickel conditions, in which hyperparameters are selected on an inner split so that no test condition informs model selection, the neural network is the most accurate at 7.6 ± 1.0 percent against 9.2 percent for the physics-anchored recovery; the support-vector and Gaussian-process baselines reach 11.2 and 11.1 percent. Within densely sampled conditions, the data-driven models are therefore the better interpolators, and we no longer claim parity there. The ordering reverses under extrapolation. Training on the conduction-dominated IN625 conditions and predicting the keyhole-dominated IN718 conditions, the physics-anchored recovery is the most accurate at 11.0 percent, against 14.5 ± 3.4 percent for the neural network, 20.0 for the support-vector model, and 23.2 for the Gaussian process. Because this comparison rests on a single train–test split with few samples, we report it as indicative. The conduction-model input supplies the keyhole trend that the training data alone never exhibit. We assess 316L separately, since it is excluded from the pooled comparison. On the 316L interpolation set, evaluated by five-fold cross-validation over its 210 conditions, the data-driven baselines reach about 20 to 21 percent against 27 percent for the physics-anchored recovery. The physics-based advantage is therefore specific to extrapolation rather than to any one alloy, and extrapolation is the regime where qualification most needs a dependable prediction.

## 4. Discussion

Treating the conduction model as an object of validation rather than of calibration alters what the comparison can reveal. Because the absorptivity is fixed to an independently determined value, every discrepancy between prediction and measurement becomes an interpretable statement about the physics-based model itself, rather than a residual that an adjustable parameter would otherwise absorb. On this footing, the model reproduces melt-pool depth and half-width together throughout the conduction regime of the largest dataset and underpredicts the depth once keyholing begins, and because this behavior recurs across three chemically distinct alloys, the limitation appears intrinsic to surface conduction rather than specific to any one material system.

The principal contribution of this work is a fitting-free diagnosis of that breakdown, and its novelty lies in three specific steps. The first is to fix the absorptivity from independent data so that the inversion tests the model instead of calibrating it. The second is to treat the non-existence of a physical solution as a signal that takes no absorptivity as input. The third is to test that signal against the principal alternative mechanism rather than argue it away. Because the unconstrained inversion draws only on the measured depth, it cannot be biased by an assumed absorptivity, and the value it returns therefore carries direct physical meaning. In the conduction regime the inversion recovers, for two independent alloys whose closed-form inputs differ by a quarter, the same physically reasonable absorptivity near 0.38. This agreement across alloys is not imposed by the method, and it shows that the forward model is sound where conduction applies. In the keyhole regime, the inferred absorptivity exceeds unity and is frequently unattainable at any value up to the search ceiling, so surface conduction cannot account for the measured depth and keyhole penetration becomes physically necessary. The diagnosis coincides with the geometric onset of keyholing, identified from the pool aspect ratio. Because that geometric limit derives from the shape a surface source can produce, the agreement is a consistency check rather than a second independent measurement; it nevertheless supplies a mechanistic reading that prior benchmarking studies, which located the breakdown without explaining it, did not provide, since the model fails precisely where the pool ceases to be a shallow conduction cap and becomes a deep, multiply reflecting cavity. A directly measured Ti-6Al-4V absorptivity, which rises by a factor of about 1.9 across the same transition, supports the assumed conduction-to-keyhole increase as a real physical mechanism rather than an artifact of the closed-form input.

A natural alternative to keyhole formation is convection-driven deepening, in which enhanced melt transport delivers the missing depth without a vapor depression. Three observations make keyholing the more parsimonious explanation. First, the above-unity absorptivity survives not only variation in the effective conductivity across its entire physical range but also an explicitly anisotropic transport enhancement. Directional advection does raise the aspect ratio a conduction description can attain, so the geometric limit is not fundamental; but at the enhancement reported for Marangoni-driven flow it resolves none of the keyhole-classified conditions, and an enhancement of ten, well outside reported values, still leaves half of them unexplained. The shortfall in the keyhole regime is therefore not closed by redistributing energy within the pool, and multiple reflections inside a cavity, which raise the absorbed fraction directly, remain the mechanism that closes it. Second, the directly measured Ti-6Al-4V coupling rises across the transition, and a rising absorptivity is the optical signature of a multiply reflecting cavity rather than of convection, which leaves the absorbed fraction unchanged. Third, the breakdown coincides abruptly with the geometric onset of keyholing rather than growing smoothly. We therefore attribute the deficit to keyholing, while not excluding a convective contribution near the threshold.

By contrast, the empirical correction exposes the limits of a purely parametric repair. An enthalpy-indexed term recovers depth within a single alloy, but its slope is material-specific, and neither a pooled enthalpy correction nor a richer multivariable descriptor transfer once a previously unseen alloy must be predicted. The same asymmetry emerges in the comparison against data-driven baselines: a flexible regressor supplied with the conduction depth is the better interpolator within densely sampled conditions, yet it degrades markedly under extrapolation between alloys, where the physics-anchored recovery is the most accurate of the four. Because qualification of new materials and parameter sets is fundamentally an extrapolation problem, the durable and transferable result of this study is the diagnosis rather than the correction. In practice, this provides an inexpensive go/no-go test for qualification: it flags, from a single measured depth and without any solver, the process conditions at which a conduction model can no longer be trusted and a keyhole-aware treatment becomes mandatory.

Several boundaries delimit these conclusions. The IN718 absorptivity is measured in situ, whereas the IN625 and 316L values are closed-form and anchored to calorimetry only in the conduction regime; the Ye scaling is moreover an empirical collapse of melt-pool data rather than a first-principles relation, so the conduction-regime agreement is corroborative rather than fully independent. The keyhole-necessity diagnosis is nevertheless independent of this choice, since it follows from the unconstrained inversion and is further supported by the measured Ti-6Al-4V rise. The source of the 316L data reports neither the location at which the width is measured nor any repeat measurement or uncertainty, so no error bars can be given for that alloy, and its width is reported for consistency only; the nominal spot diameters of the same dataset are set by defocusing, and their beam convention is unstated, which the sensitivity analysis of Section 3.4 addresses directly. The IN625 conduction conditions use the most strongly defocused spots in the study and are reproduced poorly for the same reason, so they are excluded from the quantitative conduction-regime claim while remaining in the diagnosis, which is insensitive to the beam convention. Finally, the enthalpy-indexed correction does not generalize across alloys; we report this as a limitation of an enthalpy-only description rather than resolve it here. The extrapolation comparison likewise rests on a single cross-alloy split (IN625 to IN718, with seven target conditions), since the two nickel alloys admit only one such direction; the quantitative margin should therefore be read as indicative, while under extrapolation the physics-based model remains clearly more accurate than the support-vector and Gaussian-process baselines and at least as accurate as the neural network. In addition, all benchmark data are single tracks on bare plates, whereas qualification ultimately concerns powder bed deposition; a powder layer alters both the absorptivity and the pool shape, and extending the diagnosis to powder bed conditions remains a subject of future work. The three alloys studied are all iron-based or nickel-based, so generalization to systems with markedly different optical and thermal behavior, such as aluminum, copper, or refractory metals, remains to be tested.

Each of these boundaries indicates a concrete extension. In situ coupling measurements for additional alloys would broaden the empirical basis of the inversion, and a more complete dimensionless descriptor may recover the depth deficit where the normalized enthalpy alone cannot. The same validation-and-inversion workflow extends naturally to multi-track and multi-layer deposition, where inter-track reheating offers a further and independent test of the conduction limit. Because the entire analysis runs on public benchmark data within a single notebook and invokes no finite-element or computational-fluid-dynamics solver, each extension can be pursued at negligible computational cost; more broadly, the diagnostic principle employed here, namely inverting an inexpensive physics-based model against a single measured quantity to localize where its physics fails, should apply well beyond laser powder bed fusion.

## 5. Conclusions

The absorptivity of a conduction model can be fixed from independent data rather than fitted, and doing so turns the model into an instrument that reports where its own physics fails. Applied to 231 single tracks across three alloys, the resulting inversion is unambiguous at both ends of the process window: no conduction-regime condition requires a non-physical absorptivity, and every keyhole-classified condition does. The inferred value in the conduction regime, 0.38 for two alloys whose closed-form inputs differ by a quarter, is physically reasonable and is not imposed by the method.

The strength of the conclusion is bounded by what was tested. Because an inferred absorptivity absorbs unmodeled transport, as well as optical absorption, the alternative of convective deepening was tested explicitly rather than argued away; a directional transport enhancement of the magnitude reported for Marangoni flow resolves part of the transition band and none of the keyhole conditions, and only an enhancement well outside reported values resolves half of them. Within the assumptions of a surface-conduction forward model, therefore, keyhole penetration is required at the geometric onset; this statement is not proof that no other mechanism contributes near the threshold. The nickel datasets comprise seven and fourteen conditions, the 316L dataset reports no measurement uncertainty and no beam convention, and all conditions are single tracks on bare substrates, so powder-layer absorptivity and layer-to-layer heat accumulation remain untested. Applying the framework to an alloy system with markedly different optical behavior therefore requires that the variation in absorptivity with energy density be verified for that system first. Set against the roughly twenty hours on sixty-four cores per track reported for high-fidelity thermo-fluid simulation, the cost of the test reported here is negligible.

Two practical consequences follow. A single measured depth, with no solver and about half a second of computation, suffices to decide whether a conduction model can be trusted at a given process point, which makes the test usable inside a qualification loop; the full 231-condition analysis runs in under two minutes on one core. And because an enthalpy-indexed correction transfers only between alloys that share a regime distribution, whereas the diagnosis transfers by construction, the durable cross-material result of this study is the diagnosis rather than the correction.

## Figures and Tables

**Table 1:** Thermophysical inputs (melting-range averages). All three alloys use the physical reference conductivity, with no calibration.

Property	IN718	IN625	316L
Liquidus temperature, T_l_ (K)	1610	1623	1675
Density, ρ (kg∙m^−3^)	8190	8440	7107
Specific heat, c_p_ (J∙kg^−1^∙K^−1^)	650	620	682
Latent heat, L_e_ (kJ∙kg^−1^)	210	227	260
Reference conductivity, k (W∙m^−1^∙K^−1^)	30	30	35.6
Ambient temperature, T_0_ (K)	297	297	297

**Table 2:** Single-track benchmark datasets. IN718 and Ti-6Al-4V have a measured absorptivity; IN625 and 316L use the closed-form value with flat-surface plateaus of 0.27 and 0.34 (see Methods). Regimes follow the source-dataset criterion on depth over full width. The 316L width is reported for consistency only, and Ti-6Al-4V supports the absorptivity input rather than the depth analysis.

Alloy	Conditions	Absorptivity Input	Varied Parameters	Regimes Covered
IN718	7 tracks	measured (in situ), 0.55–0.73	P: 245–325W, V: 800~1200 mm/s, D4σ: 49~82 μm	Transition, keyhole
IN625	14 tracks	closed-form, *A*_0_ = 0.27	D4σ: 50~256 μm at 180, 195 W	Conduction, transition, keyhole
316L	210 tracks	closed-form, *A*_0_ = 0.34	P: 50–500 W, V: 300~1500 mm/s, D4σ: 50~140 μm	Conduction, transition, keyhole
Ti-6Al-4V	2 modes	measured (sphere)	Stationary 102 W, scanned 196 W, 700 mm/s	Conduction and keyhole

**Table 3:** Fitting-free prediction error with the fixed absorptivity, as the mean signed percentage error within each regime (distinct from the leave-one-out MAPE in Section 3.5 and Section 3.6). A positive value is an overprediction. IN718 contains no conduction-regime condition.

Alloy	Regime	Depth Error (%)	Half-Width Error (%)	Mean Deficit (µm)
IN718	Transition	−37.2	+15.4	41.0
IN718	Keyhole	−52.4	+30.6	95.0
IN625	Conduction	−33.0	+40.1	11.5
IN625	Transition	−42.2	−10.3	37.5
IN625	Keyhole	−54.1	+31.4	79.9
316L	Conduction	+1.5	+1.1	−0.6
316L	Transition	−24.4	+7.4	34.0
316L	Keyhole	−56.2	+27.1	145.8

**Table 4:** Fraction of 316L tracks needing an above-unity absorptivity under property perturbations, resolved by regime.

Perturbation	Conduction (*n* = 132)	Transition (*n* = 60)	Keyhole (*n* = 18)
Baseline	0%	32%	100%
Conductivity +10%	0%	32%	100%
Conductivity −10%	0%	35%	100%
Specific heat +10%	0%	42%	100%
Liquidus +5%	0%	38%	100%

**Table 5:** Per-alloy depth recovery by the enthalpy correction (leave-one-out mean absolute percentage error). The pooled row is the error over all 231 conditions.

Alloy	Tracks	*δ(β)* Slope (µm)	*r*	Baseline Error	Pooled Fit	Per-Alloy Fit
IN718	7	1.31	0.979	45.9%	13.1%	4.9%
IN625	14	0.944	0.987	37.3%	29.4%	8.0%
316L	210	0.648	0.486	19.5%	26.8%	25.5%
Pooled	231	0.672	0.513	21.4%	26.5%	23.8%

**Table 6:** Depth error (mean absolute percentage) of the physics-anchored recovery versus data-driven baselines on the nickel alloys. Interpolation uses nested leave-one-out cross-validation with hyperparameters selected on an inner split; the modal selection is listed. The neural-network values are the mean and standard deviation over eight random initializations; the other three models are deterministic.

Model	Interpolation (Nested LOO)	Extrapolation (IN625 → IN718)	Selected Hyperparameters
Hybrid *δ(β)*	9.2%	11.0%	Linear in *β*, 1 degree of freedom
Support vector	11.2%	20.0%	*C* = 10, γ = 0.1, ε = 0.01
Neural network	7.6 ± 1.0%	14.5 ± 3.4%	(32, 32), α = 0.01
Gaussian process	11.1%	23.2%	RBF (l=1) + White (0.1)

## Data Availability

The original contributions presented in this study are included in the article. Further inquiries can be directed to the corresponding author. All datasets analyzed in this study are publicly available. The IN718 single-track depth, width, and in situ laser-coupling measurements are from the NIST AM-Bench AMB2022-03 dataset (https://doi.org/10.18434/mds2-2716). The IN625 single-track cross-sectional geometry is from the associated NIST datasets (https://doi.org/10.18434/mds2-2923 and https://doi.org/10.18434/mds2-3830). The 316L single-track data are from the single-source dataset of Hofmann et al. (https://doi.org/10.1016/j.matdes.2026.115459). The Ti-6Al-4V absorptance data are from the NIST A-AMB2022-01 dataset (https://doi.org/10.18434/mds2-2525). The IN625 and 316L absorptivities are computed from the closed-form scaling of Ye et al. and cross-checked against bare-plate calorimetry. All derived quantities can be regenerated from these sources using the analysis notebook described under code availability. The complete analysis is provided as a single Python 3.8 notebook that reproduces every figure and table from the public data listed above and uses no finite-element or computational-fluid-dynamics solver. The notebook will be deposited in a public repository link: https://github.com/gisukhong/AM (accessed on 31 July 2026).
